# Clinical Society of Manchester

**Published:** 1888-03

**Authors:** 


### THE CLINICAL SOCIETY OF MANCHESTER, ENGLAND.

*Etiology of Pelvic Disease in Women.*—Dr. Le Page read a paper on the etiology of those pathological conditions in woman which had their origin in the pelvis. The principal points in which woman differed physiologically from other females were considered. In the lower animals, the rut was short, the anti-rut long—that is, sexual activity was of short duration; sexual repose was prolonged. In woman, there was no interval during which the sexual passion was in abeyance. The life of a woman naturally divided itself into three periods: 1, the pre-menstrual; 2, the menstrual; 3, the post-menstrual; and many of the diseases of women may be traced to the first period, the whole of which was occupied in the development of the organs of sex. The causes of pelvic disease, operating in the period bounded by the maturation of the sexual organs and the decadence of generative functional activity, were then reviewed.

*Hydronephrosis.* — Mr. Bishop showed the kidney of a patient suffering from hydronephrosis, which he had removed a month before; also, a microscopical section of the degenerated renal tissue, showing the atrophied Malpighian tufts and tubules. The operation was post-peritoneal and extracapsular. Some collapse followed, but the patient made a good recovery.

